# Clinical Impact of Total Neoadjuvant Therapy Combined with Dose-Escalated Intensity-Modulated Radiotherapy for Lower Rectal Cancer: A Comparison with Conventional Neoadjuvant Chemoradiotherapy

**DOI:** 10.3390/cancers18132117

**Published:** 2026-06-30

**Authors:** Mikio Kawamura, Yutaka Toyomasu, Shinji Yamashita, Hideharu Ieki, Hiroki Imaoka, Tadanobu Shimura, Takahito Kitajima, Yoshinaga Okugawa, Yoshiki Okita, Masaki Ohi, Yoshihito Nomoto, Yuji Toiyama

**Affiliations:** 1 Department of Gastrointestinal and Pediatric Surgery, Mie University, 2-174 Edobashi, Tsu 514-8507, Mie, Japan; shinji-y@med.mie-u.ac.jp (S.Y.); h-ieki@med.mie-u.ac.jp (H.I.); h-imaoka@med.mie-u.ac.jp (H.I.); t-shimura@med.mie-u.ac.jp (T.S.); nyokkin@med.mie-u.ac.jp (Y.O.); mohi1012@med.mie-u.ac.jp (M.O.); ytoi0725@med.mie-u.ac.jp (Y.T.); 2 Department of Radiology, Mie University Hospital, Mie University, 2-174 Edobashi, Tsu 514-8507, Mie, Japan; y-toyomasu@med.mie-u.ac.jp (Y.T.); nomoto-y@med.mie-u.ac.jp (Y.N.); 3 Department of Genomic Medicine, Mie University Hospital, Mie University, 2-174 Edobashi, Tsu 514-8507, Mie, Japan; t-kitajima@med.mie-u.ac.jp (T.K.); yosinaga@med.mie-u.ac.jp (Y.O.)

**Keywords:** locally advanced rectal cancer, neoadjuvant, intensity modulated radiotherapy, complete response

## Abstract

**Background**: This study aimed to compare the outcomes and safety profiles of total neoadjuvant therapy (TNT) combined with dose-escalated intensity-modulated radiotherapy (IMRT) and conventional neoadjuvant chemoradiotherapy (NACRT) using three-dimensional conformal radiotherapy (3D-CRT) for locally advanced lower rectal cancer. **Methods**: This single-institution retrospective study included 51 patients treated with TNT using IMRT from 2018 to 2023 and 53 patients treated with conventional NACRT from 2001 to 2018. TNT consisted of IMRT at a total dose of 54 Gy with capecitabine, whereas NACRT consisted of 3D-CRT at a total dose of 45 Gy with a 5-fluorouracil-based regimen. Survival outcomes, treatment compliance, adverse events, surgical complications, and combined complete response (CR), defined as clinical CR plus pathological CR, were evaluated. **Results**: The combined CR rate was significantly higher in the TNT with IMRT group than in the NACRT group (43.1% vs. 5.7%). Overall survival and local recurrence-free survival did not differ significantly between the groups, whereas recurrence-free survival was significantly better in the TNT with IMRT group. Treatment compliance was comparable between the groups. Grade ≥ 3 adverse events during radiotherapy were less frequent in the TNT with IMRT group than in the NACRT group (7.8% vs. 22%), and major surgical complications did not differ significantly. **Conclusions**: TNT combined with dose-escalated IMRT was associated with a higher combined CR rate, favorable disease-free survival, and acceptable toxicity in this cohort. However, given the retrospective historical control design, chronological differences, follow-up imbalance, and potential residual confounding, these findings should be interpreted cautiously and require validation in prospective studies with longer follow-up.

## Simple Summary

Preoperative chemoradiotherapy for lower locally advanced rectal cancer has traditionally used three-dimensional conformal radiotherapy (3D-CRT) with 45–50 Gy radiation. However, strategies involving dose escalation and improved techniques have been explored to enhance efficacy. Intensity-modulated radiation therapy (IMRT) allows safer dose escalation and may improve tumor response. Although total neoadjuvant therapy (TNT) has displayed survival benefits and the potential for organ preservation, the optimal radiation approach with TNT remains uncertain. In this study, we found that TNT with IMRT at 54 Gy was associated with better tumor responses and survival rates compared with conventional NACRT with 45 Gy via 3D-CRT, without increased toxicity. Although data on TNT using dose-escalated IMRT are still limited, this approach appears to be a promising treatment option.

## 1. Introduction

Neoadjuvant therapy for locally advanced rectal cancer (LARC) has undergone a dramatic transformation with the advent of total neoadjuvant therapy (TNT), making a great paradigm shift. In various clinical trials, TNT has shown the potential to improve patient prognosis compared with conventional neoadjuvant chemoradiotherapy (NACRT) followed by surgery and has enabled non-operative management (NOM) with its high response rate [1,2,3,4]. However, the optimization of the chemotherapeutic and radiotherapeutic components of TNT remains under eager investigation.

The standard neoadjuvant radiotherapy technique used for LARC was three-dimensional confocal radiotherapy (3D-CRT), containing a total dose of 45–50.4 Gy delivered in 25–28 fractions, as recommended in several guidelines [5,6]. Since conventional NACRT was the mainstay, numerous strategies have been investigated for radiation therapy, such as optimizing concurrent chemotherapy [7,8,9] and increasing radiation doses [10]. Recently, the innovative radiotherapy technique known as intensity-modulated radiotherapy (IMRT) has led to its trial implementation for treating LARC [11,12,13]. In 3D-CRT, the radiation dose is uniform within each treatment field; however, in IMRT, complex computer-controlled manipulation of the multileaf collimator allows modulation of radiation intensity within the field. By combining such modulated beams from multiple directions, an ideal beam pattern can be created. As a result, the dose to organs at risk (OARs) can be reduced, while the dose to the target can be escalated. Because this approach is expected to simultaneously enhance histological effects and reduce radiation-related adverse events, its application for treating rectal cancer has been explored [12,14]. In addition, previous studies have shown that radiation dose escalation may also increase the chance of a pathological complete response (pCR) [10,14,15,16,17].

At present, reports on TNT with dose escalation using IMRT remain very limited. To address this, we conducted a comparative evaluation of treatment outcomes between LARC patients who underwent TNT with dose-escalated IMRT and those who received conventional NACRT using 3D-CRT. In this study, we aimed to assess and compare the complete response rates, safety profiles, and survival outcomes associated with these therapeutic approaches.

## 2. Materials and Methods

This study is a retrospective observational study using a prospective database on all patients treated consecutively between 2001 and 2023. In our institution, NACRT was introduced in 2001, with TNT using IMRT initiated in 2018. We compared treatment outcomes between two LARC patient groups: consecutive cases treated with NACRT from 2001 to 2018 and cases treated with TNT using IMRT from 2018 and later. This study was approved by the Ethics Committee of Mie University Hospital (approval number: H2025-058). Because of the retrospective nature of this study, the Ethics Committee of the Mie University Hospital waived the requirement of informed consent from the patients.

### 2.1. Sample Population, Inclusion Criteria, and Exclusion Criteria

The inclusion criteria for this study were patients aged 18 years or older with LARC (cT2–4 and/or node-positive) who were pathologically diagnosed with rectal adenocarcinoma. All patients underwent a comprehensive preoperative evaluation using blood tests, including tumor marker assessments, magnetic resonance imaging (MRI), computed tomography (CT), and colonoscopy, conducted by multiple specialists in each respective field. Patients with suspected distant metastases, a history of pelvic irradiation, or synchronous malignancies were excluded from the study.

### 2.2. Treatment Protocol

#### 2.2.1. Neoadjuvant Chemotherapy

**Figure 1:**
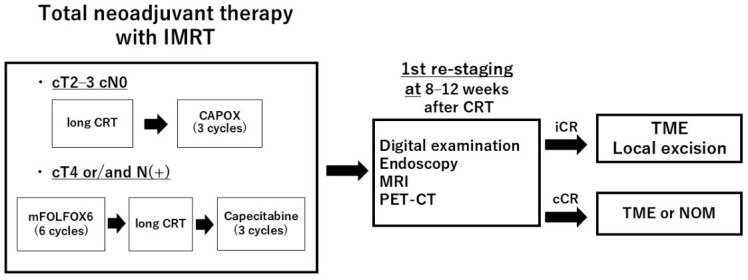
Treatment protocol in the total neoadjuvant therapy group. Patients received total neoadjuvant therapy with IMRT followed by re-staging at 8–12 weeks after CRT to determine subsequent management. Abbreviations: IMRT, intensity-modulated radiation therapy; CRT, chemoradiotherapy; CAPOX, capecitabine plus oxaliplatin; mFOLFOX6, modified fluorouracil, leucovorin, and oxaliplatin; MRI, magnetic resonance imaging; PET-CT, positron emission tomography-computed tomography; iCR, incomplete clinical response; cCR, complete clinical response; TME, total mesorectal excision; NOM, non-operative management.

In the TNT group, for patients with cT4 and/or clinically node-positive disease, six cycles of modified fluorouracil, leucovorin, and oxaliplatin (mFOLFOX6) were administered as induction chemotherapy, followed by chemoradiotherapy (CRT) and three cycles of capecitabine (Figure 1). Each mFOLFOX6 cycle consisted of oxaliplatin (85 mg/m^2^) and leucovorin (200 mg/m^2^) administered as a 2 h intravenous infusion, followed by a bolus injection of 5-fluorouracil (400 mg/m^2^) and a continuous infusion of 5-fluorouracil (2400 mg/m^2^) over 46 h. This regimen was repeated every 14 days. Patients received oral capecitabine at a dose of 1250 mg/m^2^ twice daily on days 1 to 14 every 3 weeks.

For patients with cT2–3 and clinically node-negative disease, CRT was followed by three cycles of capecitabine plus oxaliplatin (CAPOX) as consolidation chemotherapy (Figure 1). Each CAPOX cycle consisted of oxaliplatin (130 mg/m^2^) administered intravenously over 2 h on day 1 and oral capecitabine (1000 mg/m^2^ twice daily) on days 1–14. The treatment cycle was repeated every 3 weeks.

In the NACRT group, neoadjuvant chemotherapy was not administered in any of the cases.

#### 2.2.2. NACRT

Planning CT with a slice thickness of 2–5 mm was performed in the spine position. Patients were instructed to hold urination for 30–60 min before simulation and each treatment session.

In the TNT group, all patients received IMRT. The gross tumor volume (GTV) included the primary tumor (GTV-P) and lymph node metastasis (GTV-N). The clinical target volumes of the primary tumor (CTV-P) and lymph node metastasis (CTV-N) were defined encompassing the GTV-P with a craniocaudal 2 cm margin along with the rectum plus a radial 0.5 cm margin and the GTV-N plus a 0.5 cm margin, respectively. Subclinical lymph nodal regions included the mesorectum, presacral, obturator, and internal iliac lymph nodal regions. The planning target volume 1 (PTV1) was defined as the CTV-P, CTV-N, and subclinical lymph nodal regions with a 0.5–1.0 cm margin, while the PTV2 was the CTV-P and CTV-N with a 0.5–1.0 cm margin. A total dose of 45 Gy in 25 fractions was delivered to PTV1, followed by an additional 9 Gy in five fractions to PTV2. In our institution, IMRT plans were generated according to predefined institutional planning constraints. The IMRT treatment plans were required to cover ≥95% of the PTV with ≥95% of the prescribed dose, with ≤2% receiving ≥110% of the prescribed dose. Small bowel dose was limited to V35Gy < 180 cc, V40Gy < 100 cc, and V45Gy < 65 cc. Bladder dose was limited to V40 < 40%. For the IMRT cohort, dose-volume parameters were extracted from the treatment planning system and summarized as achieved doses. The concurrent chemotherapy regimen consisted of capecitabine (825 mg/m^2^ every 12 h on all days that radiotherapy was delivered).

In the NACRT group, all patients received a total dose of 45 Gy in 25 fractions with 3D-CRT. The PTV included the primary tumor, mesorectum, presacral, obturator, and internal iliac lymph nodal regions. The concurrent chemotherapy regimen included the following: 5-fluorouracil (425 mg/m^2^) and leucovorin (20 mg/m^2^) on the first and last 3 days of radiation therapy, capecitabine (825 mg/m^2^) every 12 h on all days that radiotherapy was delivered, or S-1 (typically administered orally for two weeks, followed by a one-week drug break, repeated for two cycles during NACRT).

Representative axial dose-distribution images of 3D-CRT and IMRT plans were also prepared to illustrate differences in dose distribution between the two techniques.

#### 2.2.3. Adjuvant Chemotherapy

If postoperative pathological lymph node metastasis was confirmed, then adjuvant chemotherapy with eight cycles of capecitabine or CAPOX was initiated within 6–8 weeks after surgery. The details of each regimen are described above.

### 2.3. Re-Staging and Decision for Surgery or NOM

In the TNT group, re-staging was performed 8–12 weeks after CRT completion. All patients underwent digital rectal examination, MRI, and sigmoidoscopy. They were evaluated according to the Memorial Sloan Kettering criteria [18] and classified as having a clinical complete response (cCR), near complete response (nCR), or incomplete response (iCR). In some cases, positron emission tomography-computed tomography (PET-CT) was performed to evaluate for metastasis. Patients with an nCR underwent repeat evaluation with endoscopy and MRI to determine a final classification as either cCR or iCR.

Patients who were classified as achieving a cCR and deemed suitable for NOM avoided surgery and were managed with a NOM approach. In contrast, patients with an iCR generally underwent total mesorectal excision (TME). For cases considered eligible for local excision (lesions ≤ 2 cm, up to ycT2 and ycN0), transanal local excision was performed.

In the NACRT group, re-evaluation with digital rectal examination, MRI, and sigmoidoscopy was performed 6–8 weeks after CRT completion. All patients in the NACRT group underwent TME.

### 2.4. Toxicity and Response Assessment

During the treatment period, patients visited every two weeks for evaluation through interviews and blood tests. All adverse events were assessed according to Common Terminology Criteria for Adverse Events (CTCAE), Version 5.0 and documented in the electronic medical records. After treatment completion, patients underwent follow-up examinations every 3 to 4 months, with CT performed every 6 months to monitor for recurrence. For patients managed with NOM, close surveillance was conducted with digital rectal examination, MRI, and sigmoidoscopy every 3 months for the first 3 years, then every 6 months thereafter. For all patients included in this study between 2001 and 2023, clinical and radiological findings were retrospectively re-reviewed and staged according to the 8th edition of the UICC TNM classification.

The pathological tumor response to preoperative therapy was classified according to the 8th edition of the American Joint Committee on Cancer (AJCC) and the College of American Pathologists (CAP) as follows: Tumor Regression Grade (TRG) 0, no residual tumor (pCR); TRG 1, minimal residual tumor; TRG 2, moderate response; and TRG 3, poor or no response(TRG1,2,3: npCR) [19].

Furthermore, as a comprehensive indicator of therapeutic efficacy, combined CR was defined, in accordance with Cercek et al., as encompassing both cases of pCR (TRG0) and those of sustained cCR persisting for more than 12 months [20]. For the surgical cases, postoperative complications and mortality within 30 days were evaluated and assessed using the Clavien–Dindo classification.

### 2.5. Statistical Analysis

Data on demographic, clinical, tumor-related, and treatment-related variables were collected from medical records. Numerical variables were compared using the Wilcoxon signed-rank test, while categorical variables were analyzed using the chi-squared test. Kaplan–Meier survival curves were constructed, with survival rates between the two groups compared using the log-rank test. Overall survival (OS) was defined as the time from treatment initiation to death from any cause. Recurrence-free survival (RFS) was defined as the interval from treatment initiation to the confirmed recurrence or distant metastasis of rectal cancer. Furthermore, local recurrence-free survival (LRFS) was evaluated, which was defined as the time from treatment initiation to the development of any type of local recurrence. As an exploratory sensitivity analysis, RFS was re-evaluated after restricting the cohort to patients who underwent radical surgery. Kaplan–Meier analysis was also performed in the subgroup of patients who underwent minimally invasive surgery. Two-sided *p*-values < 0.05 were considered statistically significant. All statistical analyses were performed using JMP Pro version 16 (SAS Institute Inc., Cary, NC, USA).

## 3. Results

### 3.1. Patient Characteristics

A total of 104 LARC cases treated between 2001 and 2023 were included. Among them, 51 patients received TNT with IMRT between 2018 and 2023, while 53 patients underwent NACRT between 2001 and 2017. The TNT with IMRT group comprised consecutive cases in which adjuvant chemotherapy was administered before and after IMRT-based CRT (Figure 1). Patients in the NACRT group were selected as consecutive cases that received 5-fluorouracil-based concurrent chemotherapy. As shown in Table 1, no significant differences in the baseline patient characteristics were found between the two groups, except for the follow-up period, which was significantly longer in the NACRT group (*p* < 0.0001).

### 3.2. Details of Neoadjuvant Chemotherapy and Adjuvant Chemotherapy (Table 2)

All 51 patients in the TNT group received preoperative chemotherapy. Of these, 30 individuals underwent induction chemotherapy, receiving chemotherapy first followed by radiotherapy, whereas the remaining 21 patients received consolidation chemotherapy, with radiotherapy administered first and chemotherapy subsequently. Induction regimens included mFOLFOX6 in 24 patients, CAPOX in one patient, and other regimens combined with molecular targeted agents in five patients. The details of these five patients, including the agents used, treatment regimens, rationale for use, and clinical outcomes, are summarized in Supplementary Table S1. All 21 patients in the consolidation group were treated with CAPOX. In the TNT group, major acute toxicities of CTCAE grade ≥ 3 related to preoperative chemotherapy were observed in six patients (11%). The most common adverse events were hematologic toxicities, which occurred in five patients (9.8%). In the subgroup analysis according to the timing of chemotherapy, treatment compliance and grade 3–4 acute toxicities were comparable between the consolidation and induction chemotherapy groups. The combined CR rate was numerically higher in the consolidation chemotherapy group, although this difference was not statistically significant (52.3% vs. 36.6%). (Supplementary Table S2). Neoadjuvant chemotherapy was not administered to any patient in the NACRT group. For adjuvant chemotherapy, seven patients (13.7%) in the TNT group and 36 patients (67.9%) in the NACRT group received treatment (*p* < 0.001).

**Table 2:** Details of perioperative chemotherapy.

Patient Backgrounds	TNT with IMRT	Conventional NACRT	*p*-Value
	*N* = 51	*N* = 53	
TNT protocols		-	
Induction chemotherapy	30 (58.8)		
Consolidation chemotherapy	21 (41.2)		
Chemotherapy regimen			
mFOLFOX6	24 (47.0)		
CAPOX	22 (43.1)		
others	5 (9.8)		
Major acute toxicity during			
neoadjuvant chemotherapy			
G3–4 overall	6 (11)		
G3–4 gastrointestinal	1 (0.9)		
G3–4 hematologic	5 (9.8)		
G3–4 others	0 (0)		
Adjuvant chemotherapy			<0.001
Yes	7 (13.7)	36 (67.9)	
No	44 (86.3)	17 (32.1)	

Abbreviations: TNT, total neoadjuvant therapy; IMRT, intensity-modulated radiation therapy; NACRT, neoadjuvant chemoradiotherapy; CAPOX, capecitabine plus oxaliplatin; mFOLFOX6, modified fluorouracil, leucovorin, and oxaliplatin; G, grade.

### 3.3. Treatment Compliance and Acute Toxicity of NACRT

The details of CRT are shown in Table 3. All patients in the TNT group received capecitabine, whereas intravenous 5-fluorouracil-based regimens were commonly used in the NACRT group, being administered to 46 (86%) patients (*p* < 0.001). The planned radiation dose was 54 Gy in the TNT group, while it was 45 Gy in the NACRT group. Temporary interruption or delay of radiotherapy occurred in one patient (1.9%) in the TNT group and in four patients (7.5%) in the NACRT group. Concurrent chemotherapy was temporarily interrupted or dose-reduced in six patients (11.7%) in the TNT group and in 11 patients (20.7%) in the NACRT group. All TNT group patients successfully completed radiotherapy, whereas treatment interruption occurred in one NACRT group patient (1.9%), although there was no significant difference between the groups.

During CRT, major acute toxicities of CTCAE grade ≥ 3 occurred in four patients (7.8%) in the TNT group and in 12 patients (22.6%) in the NACRT group, with a significantly higher incidence in the NACRT group (*p* = 0.032). In particular, grade ≥ 3 gastrointestinal toxicities were observed in seven NACRT group patients, all of which were diarrhea. Representative dose-distribution images demonstrated a more conformal dose distribution with IMRT than with 3D-CRT, particularly around the pelvic organs at risk (Supplementary Figure S1). The achieved doses of PTV, small bowel, and bladder among IMRT cohort are summarized in Supplementary Table S3.

### 3.4. Surgery, Pathological Findings, and Postoperative Complications

Table 4 summarizes the treatment strategies and outcomes after completion of neoadjuvant therapy. The median interval from CRT completion to subsequent treatment was 17.1 weeks in the TNT group and 8.1 weeks in the NACRT group (*p* < 0.001). In the TNT group, 32 patients (62.8%) underwent surgery, while 19 patients (37.2%) were managed with NOM. In contrast, all patients in the NACRT group proceeded to surgery.

The following analysis of the TNT group is limited to the 32 surgical cases (excluding the 19 patients managed with NOM). Minimally invasive approaches, including laparoscopic and robotic surgery, were employed in 28 of 32 patients (84.3%) in the TNT group, which was significantly higher compared with 15 patients (28.1%) in the NACRT group (*p* < 0.001).

In the NACRT group, more than 50% of patients had a pathological T3 stage, whereas this proportion was markedly reduced to 25% in the TNT group. For pathological N stage, the proportion of N0 patients was higher in the TNT group, although the difference was not statistically significant. For TRG, analysis was performed on the 32 surgical cases in the TNT group. Although the difference was not statistically significant, pCR (TRG 0) was observed in 12.5% of the TNT group and 5.7% of the NACRT group. Poor response (TRG 3) was noted in 6.2% of the TNT group and 24.5% of the NACRT group.

No significant differences were found between the two groups in the number of harvested lymph nodes or R0 resection rate. Although no significant difference was observed in vessel invasion, lymphatic invasion was significantly less frequent in the TNT group than in the NACRT group (3.1% vs. 32%, *p* = 0.0015). Similar rates of Clavien–Dindo grade ≥ 3 postoperative complications occurred between the two groups, with five of 32 TNT group patients (15.6%) and 10 of 53 NACRT group patients (18.8%) having them. Details of other complications are provided in Table 4, none of which showed significant differences between the groups.

### 3.5. Clinical CR, Pathological CR, and Combined CR

The CR statuses of the TNT and NACRT groups are summarized in Table 5. In the TNT group, cCR was achieved in 19 patients (37.2%). Of these 19 cases, one experienced local regrowth within one year after being assessed. Cases that developed regrowth within one year were concurrently associated with distant metastases and were therefore categorized as both local and distant failure. Therefore, the rate of successful cCR was 18 cases (35.2%). Among the two patients who developed local regrowth in the NOM subgroup, the only case occurring within one year was accompanied by synchronous distant metastases. The other case was isolated local regrowth at 18 months and was treated with salvage surgery. The clinical details and failure patterns of these two patients who developed regrowth during NOM are summarized in Supplementary Table S4. In contrast, because all patients in the NACRT group underwent surgery following neoadjuvant treatment, no cCR cases were observed. Furthermore, pCR was observed in four of the 32 surgical cases (7.8%: 4/51 cases) in the TNT group and in three of 53 cases (5.7%) in the NACRT group, but this difference was not statistically significant. When successful cCR and pCR were combined, the overall combined CR rate was significantly higher in the TNT group (22 patients, 43.1%) than in the NACRT group (three patients, 5.7%) (*p* < 0.0001).

### 3.6. Survival Analysis

**Figure 2:**
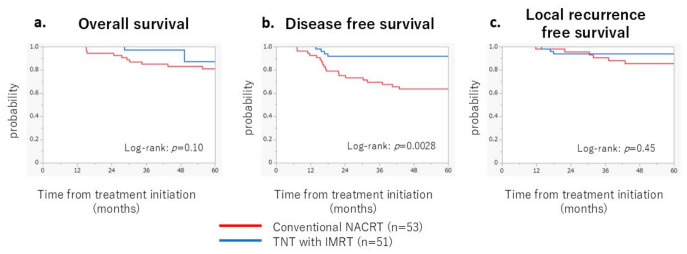
Kaplan–Meier curves for (**a**) overall survival, (**b**) disease-free survival, and (**c**) local recurrence-free survival in LARC patients treated with conventional NACRT and TNT with IMRT. Survival was calculated from treatment initiation. *p*-values were determined using the log-rank test. Abbreviations: LARC, locally advanced rectal cancer; NACRT, neoadjuvant chemoradiotherapy; TNT, total neoadjuvant therapy; IMRT, intensity-modulated radiation therapy.

Survival outcomes were compared at a median follow-up of 34.5 months in the TNT group and 74.2 months in the NACRT group. Although the shorter follow-up period in the TNT group limits the analysis to short-term outcomes, OS did not significantly differ between the two groups (Figure 2a). Four recurrence cases were observed in the TNT group, while 23 were seen in the NACRT group. In addition, among the recurrence cases in the TNT group, there were two instances of local regrowth during NOM. Consequently, the rate of local regrowth during NOM was two of 19 cases (10.5%). In both groups, the lung was the most common site of metastatic recurrence, occurring in 11 and four patients, respectively (Table 6). RFS was significantly more favorable in the TNT group compared with the NACRT group (Figure 2b; *p* = 0.0028). To reduce the potential influence of follow-up imbalance, an additional Kaplan–Meier analysis was performed with follow-up truncated at 36 months. In this restricted follow-up analysis, the TNT group continued to show significantly better RFS than the NACRT group (log-rank *p* = 0.0059). The 2-year RFS rates were 92.1% in the TNT group and 75.1% in the NACRT group, and the 3-year RFS rates were 92.1% and 69.3%, respectively (Supplementary Figure S2). In contrast, no significant difference in LRFS was observed between the TNT and NACRT groups (Figure 2c).

To reduce the potential impact of biases inherent in this retrospective historical control study, we performed exploratory subgroup and sensitivity analyses for RFS. We first re-evaluated RFS after restricting the cohort to patients who underwent radical surgery to account for baseline differences in surgical approach. In this surgically treated cohort, the TNT group showed significantly better RFS than the NACRT group, consistent with the trend observed in the overall analysis (log-rank *p* = 0.0084; Supplementary Figure S3a). When the analysis was further limited to patients who underwent minimally invasive surgery, the TNT group also showed significantly better RFS than the NACRT group (log-rank *p* = 0.034; Supplementary Figure S3b). In an additional subgroup analysis according to the timing of chemotherapy, Kaplan–Meier analysis showed no significant difference in RFS between the consolidation and induction chemotherapy groups (Supplementary Figure S4). Finally, after excluding the five patients who received molecular targeted agents, the RFS trend remained consistent with the primary analysis, suggesting that their inclusion did not substantially affect the main RFS findings (Supplementary Figure S5).

## 4. Discussion

In the present study, dose-escalated NACRT delivered using IMRT was associated with a high combined CR rate. Notably, despite escalation of the radiation dose to 54 Gy, both the treatment completion rate and incidence of adverse events were similar to those observed with conventional NACRT, suggesting an acceptable safety profile and treatment feasibility. For oncologic outcomes, although OS and LRFS displayed no significant differences, RFS was significantly improved in the TNT group. Although careful consideration is required due to the marked difference in follow-up duration, survival analyses restricted to 2- and 3-year follow-up periods showed similar results for RFS. These findings are generally consistent with those of previously reported clinical trials evaluating TNT.In major TNT trials, including STELLAR, RAPIDO, and PRODIGE 23, the reported pCR rates have generally ranged from approximately 22% to 28% [1,3,4]. In contrast, the present study demonstrated a notably higher combined CR rate of 43.1%, comprising successful cCR in 18 patients (35.2%) and pCR in four patients (7.8%). Although the inclusion of relatively low-risk and less advanced rectal cancer cases in our cohort may have partially contributed to this favorable outcome and cannot be excluded as a confounding factor, it is plausible that escalating the radiation dose to 54 Gy itself played a substantial role in enhancing treatment efficacy.

In routine clinical practice involving TNT, both the National Comprehensive Cancer Network (NCCN) and European Society for Medical Oncology (ESMO) guidelines recommend delivering 45–50.4 Gy in 25–28 fractions, with multiple clinical trials conducted under the premise that TME would be performed following neoadjuvant treatment [5,6]. This dosing regimen and treatment sequence have been established as a well-balanced strategy in terms of safety and therapeutic efficacy. However, in patients with low rectal cancer, TME is associated with substantial quality-of-life concerns, including the need for a permanent stoma following abdominoperineal resection and development of low anterior resection syndrome after sphincter-preserving surgery [21]. After Habr-Gama and colleagues first reported favorable outcomes using a watch-and-wait strategy after NACRT with a dose of 50.4 Gy in 2004, interest in NOM following neoadjuvant treatment has increased [22]. Furthermore, in 2013, the same group demonstrated that extended NACRT with dose escalation to 54 Gy enabled avoidance of TME in approximately 50% of patients with cT2–4N0–2 rectal cancer, suggesting a potential dose–response relationship between radiation dose and organ preservation [23].

Prior to the introduction of TNT, our institution routinely employed long-course CRT for rectal cancer [24]. Although pCR rates of approximately 10% to 15% have generally been reported with a radiation dose of 45 Gy [8,25,26], our institutional pCR rate with conventional CRT was only 5.7%, which was considered suboptimal. Considering this background and aiming to improve histopathological tumor responses, we adopted a dose-escalated radiation strategy consisting of 45 Gy delivered to the target volume including regional lymph nodes, followed by an additional 9 Gy boost to the primary tumor and lymph node metastases.

In designing a TNT regimen incorporating dose-escalated CRT, potential increases in treatment-related toxicities associated with higher radiation exposure to OARs, particularly gastrointestinal and urinary toxicities, were a major concern [14,15]. However, accumulating evidence has demonstrated that IMRT significantly reduces radiation doses to OARs and provides a superior safety profile compared with 3D-CRT in both rectal and prostate cancers [27,28,29]. From these data, we implemented an IMRT-based, dose-escalated CRT strategy within the TNT framework.

Using this strategy, the resulting incidence of grade ≥ 3 adverse events during the CRT phase was 7.8% in the TNT group compared with 22% in the conventional NACRT group. When adverse events related to preoperative chemotherapy were included, the overall incidence of grade ≥ 3 adverse events throughout the entire treatment course was 17.6% in the TNT group. Although the lower incidence of grade ≥ 3 adverse events in the TNT group cannot be attributed solely to IMRT, given potential period effects and residual confounding, the use of IMRT may have been associated with this reduction. The incidence of grade ≥ 3 adverse events during the preoperative treatment period in recent TNT clinical trials has been reported to range from approximately 24.2% to 47.6%, whereas corresponding rates in the NACRT arms have ranged from 12.6% to 30% [1,2,3,4]. Although radiotherapy-specific toxicity has not been clearly delineated in most TNT trials, the favorable toxicity profile observed in our TNT cohort suggests good tolerability of IMRT-based dose escalation [1,3,4]. The representative dose-distribution images showed that IMRT achieved a more conformal dose distribution than 3D-CRT, with reduced extension of intermediate- to high-dose regions into surrounding pelvic organs. Furthermore, the achieved dose-volume parameters in the current IMRT protocol indicated acceptable exposure to the small bowel and bladder according to our institutional planning constraints. These findings support the potential dosimetric advantage of IMRT and may partly explain the lower rate of severe acute adverse events in the TNT-IMRT cohort. In addition, subgroup analysis within the TNT cohort showed comparable toxicity profiles between the consolidation and induction chemotherapy groups, with no significant differences in treatment interruption or delay, chemotherapy completion, or grade 3–4 acute toxicities. The combined CR rate was numerically higher in the consolidation group than in the induction group (52.3% vs. 36.6%), although this difference was not statistically significant. This trend may reflect both the potential favorable response associated with consolidation chemotherapy [2,30] and the lower baseline stage of patients in the consolidation group under our institutional protocol. No significant difference in RFS was observed between the two groups, although longer follow-up is needed for definitive interpretation.

Since 2021, both 3D-CRT and IMRT have been used as radiation delivery techniques in TNT-related clinical trials [1,3,4]. While randomized trials have generally reported comparable local recurrence rates between TNT and NACRT, the RAPIDO trial uniquely demonstrated a higher local recurrence rate in the TNT arm [4]. Subsequent post hoc analyses revealed that this increase was confined to patients treated with 3D-CRT, with no difference in local or regional recurrence observed between TNT and NACRT among patients treated with IMRT or volumetric modulated arc therapy [31]. These findings suggest that IMRT may offer superior local control compared with 3D-CRT in the context of TNT. Consistently, the STELLAR trial, in which all patients received short-course radiotherapy delivered using IMRT, reported favorable local control and concluded that routine use of IMRT in rectal cancer treatment is feasible [1].

One of the principal advantages of TNT is the potential for organ preservation through NOM. In the OPRA trial, which focused on organ preservation, IMRT with dose escalation to 54–56 Gy was recommended, with favorable local control and high organ preservation rates achieved [2]. Moreover, analyses from the International Watch & Wait Database have demonstrated that radiation doses below 50.4 Gy are associated with higher rates of early local recurrence [32]. Considering these data, using a 54-Gy radiation dose in our study represents a rational dose-escalation strategy for TNT aimed at facilitating NOM. In our cohort, successful CR was achieved in 18 patients (35.2%), with local regrowth occurring in only two patients (10.5%) among those managed with NOM. These outcomes are comparable to those in a previously reported NOM series [2,32,33]. Conversely, excessive dose escalation is not acceptable, as postoperative complication rates have been reported to increase at doses exceeding 58.9 Gy [14]. In our study, although careful consideration is required because of temporal differences in surgical procedures and perioperative management, surgical outcomes after TNT were comparable to those after conventional NACRT. Taken together, the available evidence supports IMRT-based TNT with a radiation dose of 54 Gy as a well-balanced preoperative treatment strategy that combines high therapeutic efficacy with acceptable tolerability, potentially contributing to improved survival outcomes and enhanced organ preservation.

This study has several important limitations. The most important limitation is its single-institution retrospective design using a historical control cohort. Because the treatment strategy at our institution completely shifted from conventional NACRT to TNT in 2018, no contemporaneous NACRT cohort was available. Therefore, patients treated before 2018 inevitably served as the comparator, resulting in a historical comparison between the pre-2018 and post-2018 treatment periods. Accordingly, the observed differences cannot be attributed solely to differences in treatment modality. Potential period effects related to improvements in imaging-based staging, surgical techniques, supportive care, and perioperative management should be carefully considered when interpreting the overall results. In addition, the follow-up duration was significantly shorter in the TNT group than in the NACRT group, and later recurrence events may not have been fully captured. Although exploratory sensitivity analyses were performed, including analyses restricted to patients who underwent radical surgery and those treated with minimally invasive surgery, residual confounding due to period effects may remain.

Another important limitation concerns the interpretation of the combined CR endpoint. NOM was not a conventional treatment option during the NACRT period, and radical surgery was generally planned regardless of clinical response. Therefore, sustained cCR could only be assessed in the TNT cohort, which may have favored the TNT group in the evaluation of combined CR. Moreover, the longer interval between radiotherapy completion and surgery or response assessment in the TNT cohort may have contributed to the higher combined CR rate [34]. Thus, although the higher combined CR rate in the TNT group is clinically meaningful, this endpoint should be interpreted cautiously in the context of historical differences in treatment strategy and response assessment.

Several additional limitations should also be acknowledged. This study did not include a direct dosimetric comparison between 3D-CRT and IMRT. Although representative dose distribution images suggest improved dose conformity with IMRT, quantitative dose–volume parameters for organs at risk were not available for both cohorts. Therefore, the contribution of IMRT to the lower incidence of severe acute adverse events should be interpreted cautiously. In addition, the TNT cohort included heterogeneity in chemotherapy sequencing and regimens. Although subgroup and sensitivity analyses showed results consistent with the primary findings, the impact of these variations cannot be completely excluded. Finally, because adverse events in the historical NACRT cohort were retrospectively reassessed from medical records, underestimation or differences in documentation quality cannot be completely excluded.

Therefore, although dose-escalated chemoradiotherapy delivered using IMRT within a TNT strategy was associated with a high combined CR rate and favorable RFS, its independent effect cannot be determined from the present study alone. The present findings should be interpreted as hypothesis-generating, and prospective studies with longer follow-up are needed to validate these results.

## 5. Conclusions

Overall, TNT incorporating dose-escalated CRT delivered with IMRT to 54 Gy was associated with a high CR rate, low systemic recurrence and local regrowth, and acceptable toxicity and treatment feasibility in patients with LARC. Although the historical comparison should be interpreted with caution because of potential temporal bias, our findings suggest that TNT combined with dose-escalated IMRT may provide favorable tolerability, encouraging oncologic outcomes, and a meaningful opportunity for organ preservation in patients with LARC.

## Figures and Tables

**Table 1:** Baseline patient and tumor characteristics.

Patient Backgrounds	TNT with IMRT	Conventional NACRT	*p*-Value
	*N* = 51	*N* = 53	
Age (years)	65 (31–82)	63 (33–83)	0.68
Gender			0.91
male	38 (74.5)	39 (73.8)	
female	13 (25.5)	14 (26.2)	
BMI	22.4 (15.8–38.9)	21.7 (16.5–30.9)	0.12
Follow up period (months)	34.5 (20.4–76.6)	74.2 (15.3–172)	<0.0001
Tumor location; distance from AV (cm)			0.23
Mid rectum (5–10)	19 (37.2)	14 (26.4)	
Low rectum (<5)	32 (62.8)	39 (73.6)	
Clinical T stage			0.10
T2	5 (9.8)	2 (3.7)	
T3	37 (72.5)	33 (62.2)	
T4	9 (17.6)	18 (33.9)	
Clinical N stage			0.095
N0	16 (31.3)	8 (15.0)	
N1	16 (31.3)	25 (47.1)	
N2	19 (37.2)	20 (37.7)	
Pathology			0.053
well, mod	49 (96.0)	45 (84.9)	
por, muc	2 (4.0)	8 (15.1)	
Pretreatment CEA ≥ 5 (ng/mL)			0.074
Yes	19 (37.2)	29 (54.7)	
No	32 (62.4)	24 (45.3)	

The data are presented as median (range) or *N* (%), unless otherwise indicated. Abbreviations: TNT, total neoadjuvant therapy; IMRT, intensity-modulated radiation therapy; NACRT, neoadjuvant chemoradiotherapy; BMI, body mass index; AV, anal verge; CEA, carcinoembryonic antigen; mod, moderately differentiated adenocarcinoma; por, poorly differentiated adenocarcinoma; muc, mucinous adenocarcinoma.

**Table 3:** Details of neoadjuvant chemoradiotherapy (NACRT), acute toxicity, and treatment compliance.

Patient Backgrounds	TNT with IMRT	Conventional NACRT	*p*-Value
	*N* = 51	*N* = 53	
Concurrent chemotherapy			<0.001
cap	51 (100)	3 (5.7)	
S-1	0	4 (7.5)	
5-FU infusion	0	46 (86)	
Planed dose of radiotherapy			-
54 Gy	51 (100)	0	
45 Gy	0	53 (100)	
Interruption or delayed CRT			0.18
Yes	1 (1.9)	4 (7.5)	
No	50 (98.1)	49 (92.5)	
Interruption or decreased			0.21
concurrent chemotherapy			
Yes	6 (11.7)	11 (20.7)	
No	45 (88.3)	42 (79.3)	
CRT completion			0.32
Yes	51 (100)	52 (98.1)	
No	0 (0)	1 (1.9)	
Major acute toxicity during CRT			
G3–4 overall	4 (7.8)	12 (22.6)	0.032
G3–4 gastrointestinal	2 (3.9)	7 (13.2)	0.09
G3–4 hematologic	3 (5.8)	6 (11.3)	0.32
G3–4 others	0	2 (3.7)	0.16
Major acute toxicity during neoadjuvant treatment	9 (17.6)	12 (22.6)	0.52

The data are presented as median (range) or *N* (%), unless otherwise indicated. Abbreviations: TNT, total neoadjuvant therapy; IMRT, intensity-modulated radiation therapy; NACRT, neoadjuvant chemoradiotherapy; CRT, chemoradiotherapy; cap, capecitabine; 5-FU, fluorouracil; G, grade.

**Table 4:** Surgery, pathological findings, and postoperative complications.

Patient Backgrounds	TNT with IMRT	Conventional NACRT	*p*-Value
	*N* = 51	*N* = 53	
Interval between CRT termination and operation or NOM (weeks)	17.1 (8–40)	8.1(4–26)	<0.001
Surgery			0.10
TME	31 (60.7)	52 (98.1)	
Local resection	1 (1.9)	1 (1.9)	
NOM	19 (37.2)	0	
**Patient Backgrounds**	***N*** ** = 32**	***N*** ** = 53**	***p*** **-Value**
Surgical approach			<0.001
Open	3 (9.3)	37 (70)	
Transanal local excision	1 (3.1)	1 (1.9)	
Minimally invasive	28 (84.3)	15 (28.1)	
Pathological T stage			0.0031
T0	5 (15.6)	3 (5.7)	
T1	10 (31.2)	3 (5.7)	
T2	9 (28.3)	16 (3.2)	
T3	8 (25)	29 (54.7)	
T4	0	2 (3.7)	
Pathological N stage			0.059
N0	29 (90.6)	36 (69.2)	
N1	2 (6.2)	14 (25)	
N2	1 (3.1)	3 (5.7)	
Tumor regression grade			0.15
0	4 (12.5)	3 (5.7)	
1	14 (43.7)	19 (35.8)	
2	12 (37.5)	18 (33.9)	
3	2 (6.2)	13 (24.5)	
Lymphatic invasion			0.0015
Yes	1 (3.1)	17 (32.0)	
No	31 (96.9)	36 (68.0)	
Vascular invasion			0.13
Yes	5 (15.6)	16 (30.1)	
No	27 (84.4)	37 (69.9)	
Harvested lymph nodes	9 (0–19)	7.5 (0–33)	0.62
R0 resection			0.59
yes	31 (96.8)	50 (94.3)	
No	1 (3.2)	3 (5.7)	
Postoperative complication (Clavien-Dindo grade ≥ 3)	5 (15.6)	10 (18.8)	0.70
Leakage anastomosis (LAR)	0	3 (5.6)	0.17
Pelvic abscess	4 (12.5)	7 (13.2)	0.92
Perineal wound dehiscence	1 (3.1)	0	0.19

The data are presented as median (range) or *N* (%), unless otherwise indicated. Abbreviations: TNT, total neoadjuvant therapy; IMRT, intensity-modulated radiation therapy; NACRT, neoadjuvant chemoradiotherapy; CRT, chemoradiotherapy; NOM, non-operative management; TME, total mesorectal excision; LAR, low anterior resection.

**Table 5:** Details of complete response (CR).

Patient Backgrounds	TNT with IMRT	Conventional NACRT	*p*-Value
	*N* = 51	*N* = 53	
Clinical CR			-
Yes	18 (35.2)	0 (0)	
No	33 (64.8)	53 (100)	
Pathological CR among surgical cases			0.26
Yes	4 (12.5)	3 (5.7)	
No	28 (87.5)	50 (94.3)	
Combined CR			<0.0001
Yes	22 (43.1)	3 (5.7)	
No	29 (56.9)	50 (94.3)	

The data are presented as median (range) or *N* (%), unless otherwise indicated. Abbreviations: TNT, total neoadjuvant therapy; IMRT, intensity-modulated radiation therapy; NACRT, neoadjuvant chemoradiotherapy; CR, complete response.

**Table 6:** Detailed recurrence sites after treatment in both TNT with IMRT and conventional NACRT (including overlapping cases).

Recurrence Patterns	TNT with IMRT	Conventional NACRT
	*N* = 51	*N* = 53
Total recurrences	4 (7.8)	23 (43.3)
Lung metastasis	4 (7.8)	13 (24.5)
Liver metastasis	1 (1.9)	4 (7.5)
Peritoneal recurrence	2 (3.8)	2 (3.7)
Local recurrence after TME	1 (1.9)	6 (11.3)
Local regrowth in NOM	2 (3.8)	–
Others	0	1 (1.8)

Data are presented as median (range) or *N* (%), unless otherwise indicated. Abbreviations: TNT, total neoadjuvant therapy; IMRT, intensity-modulated radiation therapy; NACRT, neoadjuvant chemoradiotherapy; NOM, non-operative management; TME, total mesorectal excision.

## Data Availability

The data supporting the findings of this study are available from the corresponding author upon reasonable request.

## Supplementary Materials

The following supporting information can be downloaded at: https://www.mdpi.com/article/10.3390/cancers18132117/s1, Figure S1: Comparison of dose distribution between three-dimensional conformal radiotherapy ((A) female, (C) male) and intensity-modulated radiotherapy ((B) female, (D) male)) plans; Figure S2: Kaplan–Meier Curves for Recurrence-Free Survival with Follow-up Truncated at 36 Months; Figure S3: (a) Kaplan–Meier curves for Recurrence-Free Survival among patients who underwent radical surgery. (b) Kaplan–Meier curves for Recurrence-Free Survival among patients who underwent MIS; Figure S4: Recurrence-Free Survival According to the Timing of Chemotherapy: Induction versus Consolidation Chemotherapy; Figure S5: Kaplan–Meier Curves for Recurrence-Free Survival After Excluding Patients Who Received Molecular Targeted Agents; Table S1: Details of patients who received molecular targeted agents; Table S2: Comparison of Acute Toxicity, Treatment Compliance, and Response Between Consolidation and Induction Chemotherapy; Table S3: Summary of dosimetric information of patients treated with IMRT; Table S4: Clinical Details of Patients Who Developed Recurrence During NOM.
