# Supplementary material for: Clinical Impact of Total Neoadjuvant Therapy Combined with Dose-Escalated Intensity-Modulated Radiotherapy for Lower Rectal Cancer: A Comparison with Conventional Neoadjuvant Chemoradiotherapy
Source: Cancers (Basel). 2026 Jun 30;18(13):2117. doi: 10.3390/cancers18132117 (PMC13359633; doi:10.3390/cancers18132117)
Supplement: Supplementary file 1 [file cancers-18-02117-s001.zip › cancers-4326542-supplementary.pdf]

Supplementary Table S1.
Details of patients who received molecular targeted agents

Case No.	age/gender	Clinical stage	TNT Regimen	Backbone chemotherapy	Molecular targeted agent	Rationale for targeted agent	Re-staging/ procedure	Pathological outcome	recurrence
1	71 y.o./male	cT4bN1M0	Induction	mFOLFOX6	Panitumumab	Conversion-intent treatment for locally advanced tumor (prostate invasion)	iCR/ Robot assisted APR	TRG1	No recurrence (45m)
2	72 y.o./male	cT4bN2M0	Induction	mFOLFOX6	Bevacizumab	Conversion-intent treatment for locally advanced tumor (prostate and levator invasion)	iCR/ Robot assisted APR	TRG1	No recurrence (40m)
3	55 y.o./male	cT4bN2M0	Induction	mFOLFOX6	Panitumumab	Conversion-intent treatment for locally advanced tumor (prostate invasion)	iCR/ Robot assisted APR	TRG0	No recurrence (36m)
4	52 y.o./female	cT3N2M0	Induction	mFOLFOX6	Bevacizumab	Conversion-intent treatment for locally advanced tumor	iCR/ Robot assisted LAR	TRG2	No recurrence (28m)
5	38 y.o./male	cT4bN2M0	Induction	mFOLFOX6	Panitumumab	Conversion-intent treatment for locally advanced tumor and lateral pelvic lymph node swelling	cCR/ NOM	-	No recurrence (26m)

Abbreviations:
TNT, total neoadjuvant therapy; mFOLFOX6, modified fluorouracil, leucovorin, and oxaliplatin; iCR, incomplete clinical response;
cCR, clinical complete response; APR, abdominoperineal resection; LAR, low anterior resection; NOM, non-operative management;
TRG, tumor regression grade.

Supplementary Table S2.

Comparison of Acute Toxicity, Treatment Compliance, and Response Between Consolidation and Induction Chemotherapy

	Consolidation chemotherapy n=21 (%)	Induction chemotherapy N=30 (%)	p-value
Concurrent chemotherapy			
cap	21 (100)	30 (100)	
Interruption or Delayed CRT			0.39
Yes	0 (0)	1 (3.3)	
Interruption or Delayed concurrent chemotherapy			
Yes	2 (9.5)	4 (13.3)	0.67
CRT completion			
Yes	21 (100)	30 (100)	-
Interval between CRT termination and operation or NOM (weeks)	19 (8- 37)	16.2(9.2-40)	0.68
Major acute toxicity during CRT			
G3-4 overall	2 (9.5)	2 (6.6)	0.70
G3-4 gastrointestinal	1(4.7)	1(3.3)	0.79
G3-4 hematologic	2 (9.5)	1 (3.3)	0.35
G3-4 others	0	0	-

Note: Data are shown as n (%) or median (range), as appropriate.
Abbreviations: CRT, chemoradiotherapy; CAPOX, capecitabine plus oxaliplatin; mFOLFOX6, modified fluorouracil, leucovorin, and oxaliplatin;
CR, complete response; G, grade; NOM, non-operative management.

	Consolidation chemotherapy n=21 (%)	Induction chemotherapy n=30 (%)	p-value
Chemotherapy regimen			<0.001
mFOLFOX6	0	24 (80)	
CAPOX	21(100)	1 (3.3)	
Others	0	5 (16.6)	
Interruption or Delayed chemotherapy			0.52
Yes	5 (23.8)	5 (16.2)	
chemotherapy completion			
Yes	21 (100)	30 (100)	-
Major acute toxicity during neoadjuvant chemotherapy			
G3-4 overall	3 (14.3)	3 (10)	0.64
G3-4 gastrointestinal	1 (4.7)	0	0.22
G3-4 hematologic	2 (9.5)	3 (10)	0.95
G3-4 others	0	0	-

	Consolidation chemotherapy N=21 (%)	Induction chemotherapy N=30 (%)	p-value
Clinical CR	9 (43)	9 (30)	0.34
Pathological CR	2 (9.5)	2 (6.6)	0.70
Combined CR	11 (52.3)	11 (36.6)	0.26

Supplementary Table S3.

Summary of dosimetric information of patients treated with IMRT

Variable	Dose-volume parameter	Value
PTV2	D95% (Gy)	53.9 (52.5-55.1)
	D2% (Gy)	58.8 (54.1-59.3)
Small bowel	Dmax (Gy)	52.6 (18.6-59)
	V45Gy (cc)	3.6 (0-107.7)
	V40Gy (cc)	7.8 (0-174.9)
	V35Gy (cc)	16.9 (0-232.2)
Bladder	Dmax (Gy)	55.7 (38.4-58.9)
	V40Gy (%)	19.3 (0-39.8)

Note: Values are presented as median (range).
Abbreviations: IMRT, intensity-modulated radiotherapy; PTV, planning target volume; D95%, dose covering 95% of the volume; D2%, near-maximum dose; Dmax, maximum dose; VxGy, volume receiving at least x Gy.

Supplementary Table S4.

Clinical Details of Patients Who Developed Recurrence During NOM

Case number	Failure pattern in NOM subgroup	Time to regrowth after NOM assessment	Salvage treatment	Outcome
**Case 1**	Isolated local regrowth (without metastasis)	18months	Salvage surgery	Peritoneal recurrence 6 months after surgery; systemic chemotherapy ongoing.
**Case 2**	Local regrowth with synchronous metastasis	6months	Systemic chemotherapy	Undergoing systemic chemotherapy

Abbreviations: NOM, non-operative management.

Supplementary Figure S1.

Comparison of dose distribution between three-dimensional conformal radiotherapy ((A) female, (C) male) and intensity-modulated radiotherapy ((B) female, (D) male) plans.

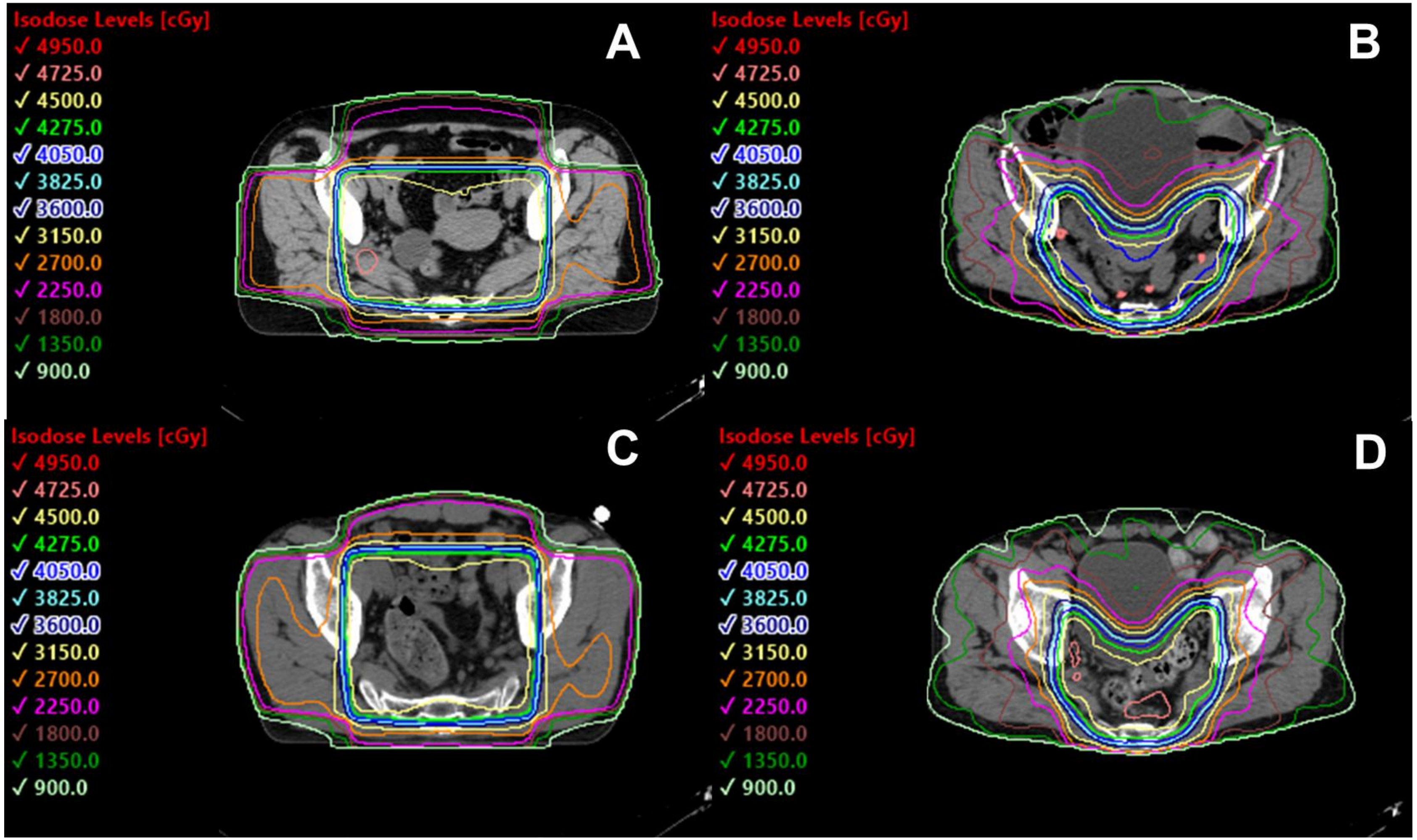

Note: Isodose levels indicate the percentage of the prescribed radiation dose delivered to each region. Higher isodose levels represent areas receiving a higher proportion of the prescribed dose.
Abbreviations: 3D-CRT, three-dimensional conformal radiotherapy; IMRT, intensity-modulated radiotherapy.

Supplementary Figure S2.

Kaplan–Meier Curves for Recurrence-Free Survival with Follow-up Truncated at 36 Months

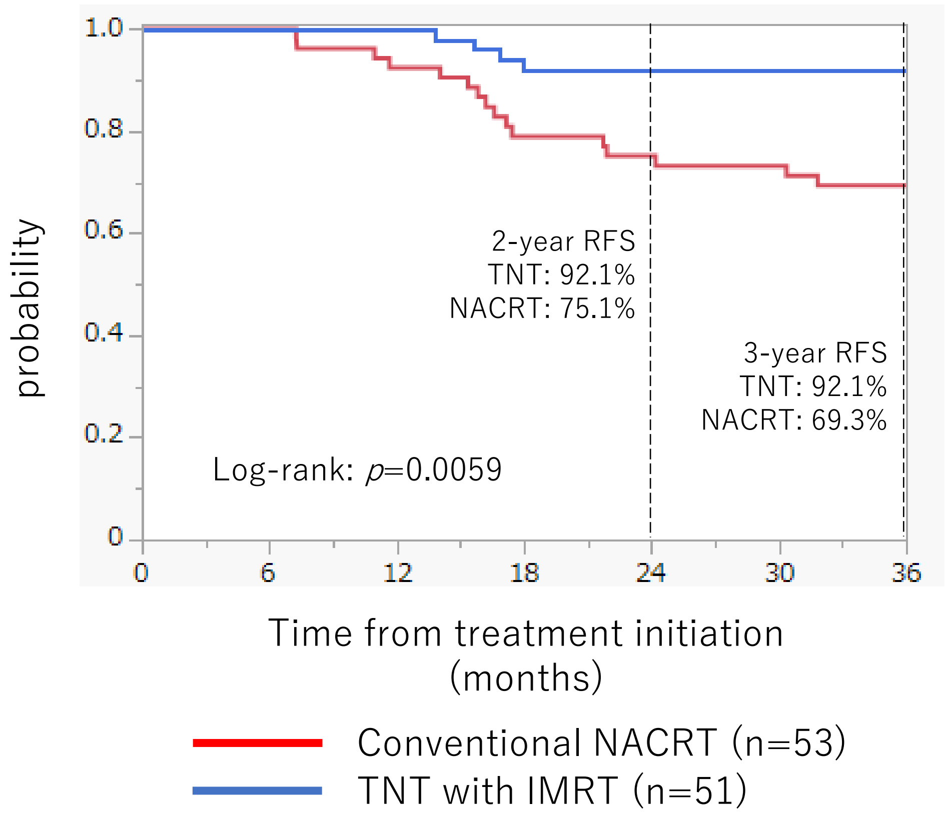

Note. The TNT group showed significantly better RFS than the NACRT group in this restricted follow-up analysis (log-rank $p = 0.0059$). The 2-year RFS rates were 92.1% and 75.1%, and the 3-year RFS rates were 92.1% and 69.3% in the TNT and NACRT groups, respectively. Abbreviations: RFS, recurrence-free survival; TNT, total neoadjuvant therapy; IMRT, intensity-modulated radiotherapy; NACRT, neoadjuvant chemoradiotherapy.

Supplementary Figure S3.

(a) Kaplan–Meier curves for Recurrence-Free Survival among patients who underwent radical surgery

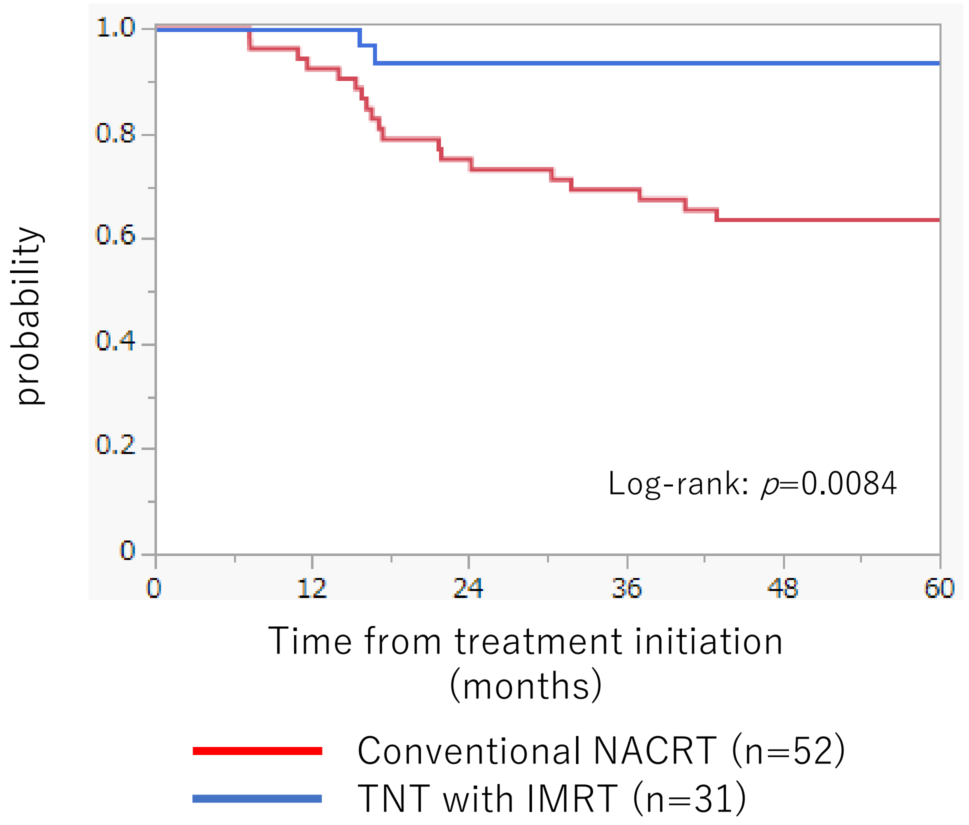

(b) Kaplan–Meier curves for Recurrence-Free Survival among patients who underwent MIS

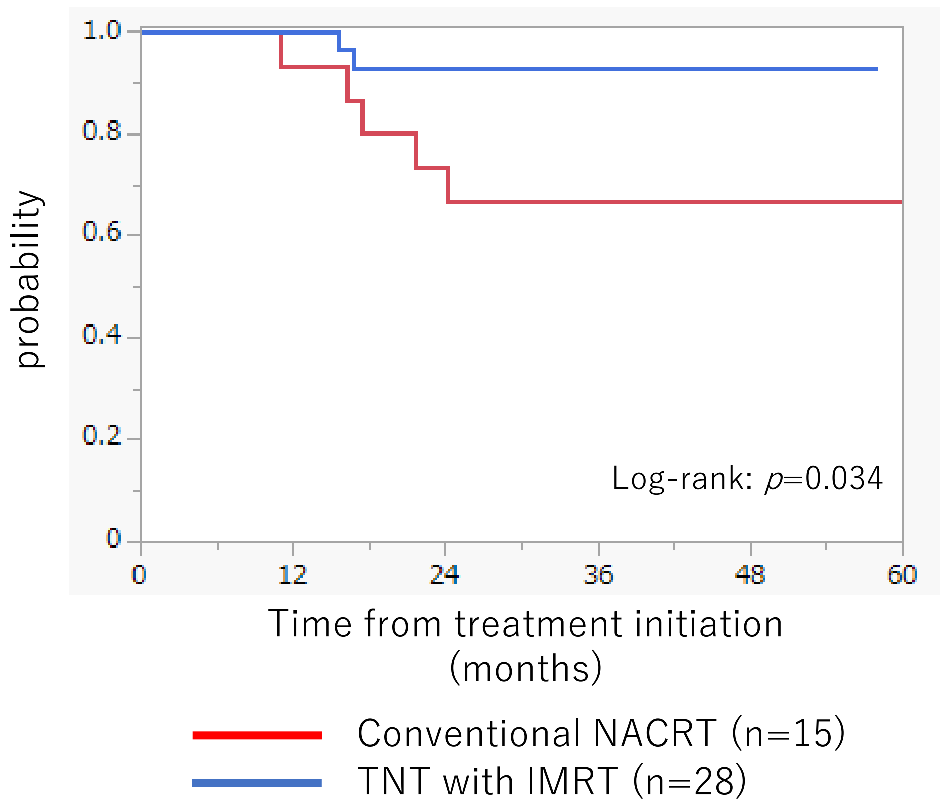

Note. (a) RFS among patients who underwent radical surgery. (b) RFS among patients who underwent minimally invasive surgery. The TNT with IMRT group showed significantly better RFS than the conventional NACRT group in both analyses.
Abbreviations: RFS, recurrence-free survival; TNT, total neoadjuvant therapy; IMRT, intensity-modulated radiotherapy; NACRT, neoadjuvant chemoradiotherapy; MIS, minimally invasive surgery.

Supplementary Figure S4.

Recurrence-Free Survival
According to the Timing of Chemotherapy:
Induction versus Consolidation Chemotherapy

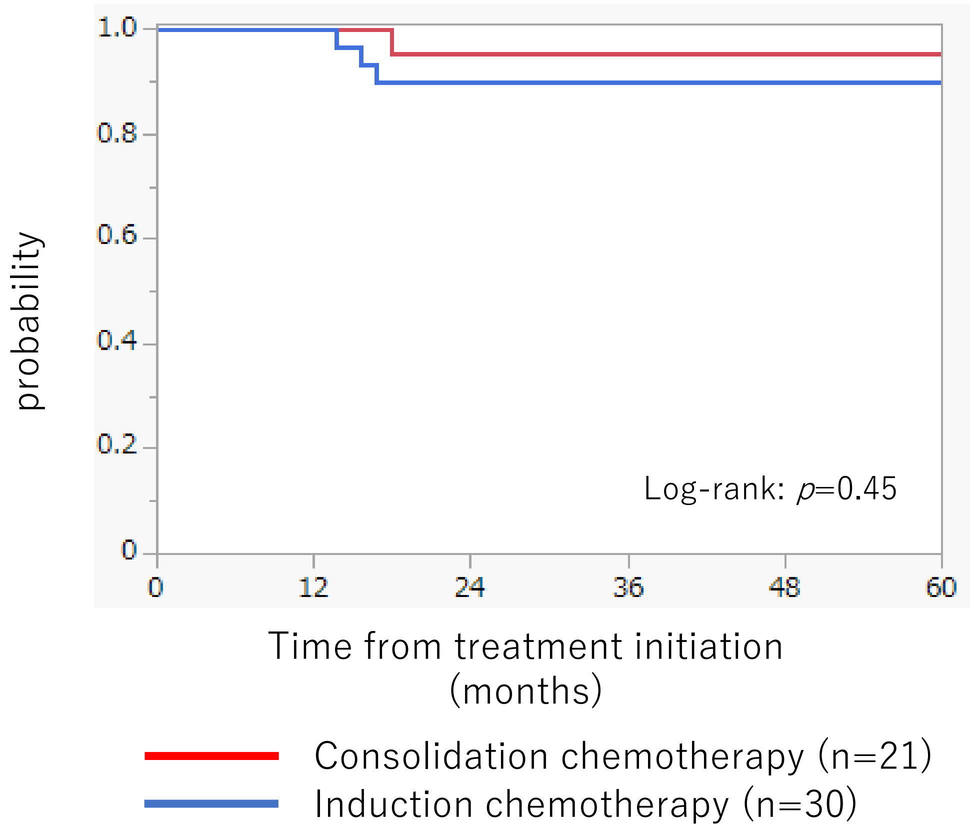

Note. Kaplan–Meier curves for RFS according to the timing of chemotherapy.
RFS was compared between patients who received induction chemotherapy and those who received consolidation chemotherapy. No significant difference in RFS was observed between the two groups.
Abbreviations: RFS, recurrence-free survival

Supplementary Figure S5.

Kaplan–Meier Curves for Recurrence-Free Survival
After Excluding Patients Who Received Molecular Targeted Agents

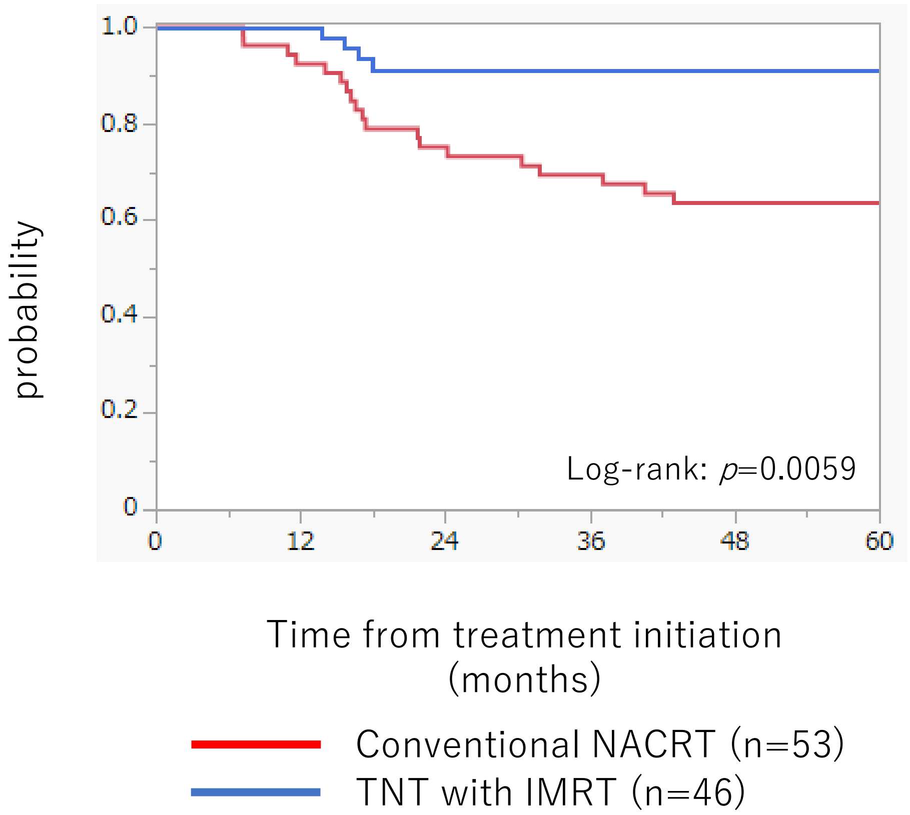

Note: Kaplan–Meier curves for recurrence-free survival after excluding the five patients who received chemotherapy combined with molecular targeted agents. This sensitivity analysis was performed to assess the potential impact of treatment heterogeneity introduced by molecular targeted agents.

Abbreviations: RFS, recurrence-free survival; TNT, total neoadjuvant therapy; IMRT, intensity-modulated radiotherapy; NACRT, neoadjuvant chemoradiotherapy.
